# The AI adaptation divide in medical education: A generational perspective

**DOI:** 10.1371/journal.pdig.0001338

**Published:** 2026-04-10

**Authors:** Nghia Phu Nguyen, Phillip Tran

**Affiliations:** College of Health Sciences, Nam Can Tho University, Can Tho City, Vietnam; University of Bremen Faculty 11 Human and Health Sciences: Universitat Bremen Fachbereich 11 Human- und Gesundheitswissenschaften, GERMANY

Artificial intelligence (AI) is no longer a distant possibility in medical education; it is already reshaping how students learn and how teachers teach [1]. While curricula, ethics, and institutional frameworks have been explored, one critical dimension remains underexamined: the generational gap in AI readiness between digital-native students and senior faculty [2,3]. In this context, AI adaptation refers to the capacity to integrate AI meaningfully into educational practice, including appropriate use, critical evaluation, and ethical judgment. This “AI adaptation divide” reflects not only disparities in technical skill but also deeper contrasts in culture, mindset, and trust. The question is no longer whether AI will enter classrooms, but whether students and educators will adapt together or grow apart along generational lines.

Existing evidence suggests that the AI adaptation divide stems from a recurring constellation of generational differences in how AI is approached, learned, and governed within medical education. Misalignments in readiness, expectations, and professional priorities between students and faculty further contribute to this divide. Table 1 summarizes these patterns by contrasting how students and faculty currently engage with, interpret, and respond to AI in educational settings.

**Table 1:** Manifestations of the artificial intelligence (AI) adaptation divide in medical education [1,4–8].

Dimension of AI adaptation	Students	Faculty
Mode of initial engagement	Begin using AI informally for studying, clarification, and productivity, often outside formal curricula	Engage later and more cautiously, often waiting for institutional guidance or formal training
Primary motivation for use	Efficiency, personalization of learning, exam preparation, and cognitive offloading	Improving teaching efficiency, assessment design, and academic productivity, while preserving educational standards
Perceived competence	Report higher confidence and familiarity, though often based on experimentation rather than formal training	Frequently self-identify as novices, expressing uncertainty about the appropriate and effective use
Approach to ethical risk	Acknowledge concerns, but tend to normalize AI as a learning aid	Emphasize risks related to academic integrity, bias, privacy, and erosion of critical thinking
Preferred learning pathway	Self-directed exploration, peer sharing, and trial-and-error	Structured workshops, formal guidelines, and expert-led training
Relationship to institutional support	Operate largely independently of institutional frameworks	Depend on institutional policies, governance, and protected time for engagement

Across these dimensions, AI adaptation takes shape through how AI is integrated, regulated, and interpreted within distinct generational roles. When these adaptive pathways diverge, the divide risks fracturing the intergenerational cohesion that sustains medical education. Without adequate preparation, faculty may be sidelined, leaving students unguided [9]. This mirrors the digital divide that shaped global education policy, but the stakes are higher in medicine: AI-trained students may graduate with fragmented practices lacking ethical oversight, while faculty risk diminished authority in classrooms [5]. These risks are likely amplified in low- and middle-income country (LMIC) settings, where limited institutional infrastructure, uneven access to faculty development, and the absence of clear governance frameworks constrain coordinated AI adoption [3,7]. In such contexts, students often engage with AI independently through informal channels, while faculty face structural barriers to training and policy support [5,7,8], widening the adaptation divide and increasing the likelihood of unsupervised, ethically inconsistent use.

**Fig 1:**
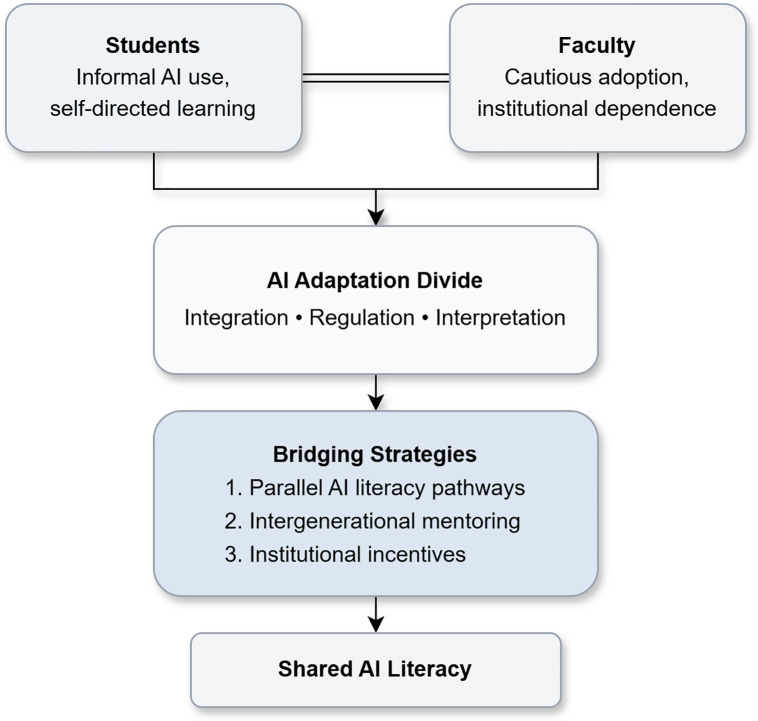
Proposed strategies to bridge the artificial intelligence (AI) adaptation divide in medical education. The figure illustrates how generational differences between students and faculty may converge toward shared AI literacy through three complementary strategies. (1) Parallel AI literacy tracks respond to divergent learning preferences, for example, by emphasizing guided workshops on ethical reasoning, clinical validation, and responsible AI use for faculty, alongside supervised, reflective AI use embedded within student coursework. (2) Intergenerational mentoring enables bidirectional exchange, in which students or junior faculty may introduce emerging AI tools or workflows, while senior educators contribute clinical judgment, contextual oversight, and ethical framing, including the interpretation of ethical and security implications in clinical contexts. (3) Institutional incentives and support include governance functions such as ethical review of AI-related educational materials, alignment with institutional policy, and oversight of co-learning initiatives, with consultation from external ethics or data security experts where appropriate.

To avoid this fragmentation, AI adaptation strategies should intentionally align with generational realities. As illustrated in Fig 1, we propose three complementary approaches to bridge the adaptation divide: parallel AI literacy tracks, intergenerational mentoring, and institutional incentives.

Among these, reciprocal intergenerational mentoring remains comparatively underexplored, despite its potential to align digital fluency with clinical and ethical expertise across generations. In much of the prevailing discourse on AI integration, adaptation is often framed in terms of faculty development or student training as parallel endeavors, instead of being seen as deliberately structured intergenerational exchange. In some training environments, existing hierarchical norms may further limit bidirectional learning, even when technical and clinical expertise are distributed across generations. Such asymmetries can discourage students from legitimizing their technical expertise and constrain faculty from engaging openly with unfamiliar tools. As a result, opportunities to integrate emerging AI practices with professional judgment and ethical oversight may be missed, reinforcing the AI adaptation divide.

The AI adaptation divide represents a fundamentally generational challenge in medical education, shaped by how adaptation efforts are currently organized. In many institutions, guidelines governing AI use are now in place, and parallel training initiatives for faculty and students have been introduced to support responsible adoption. As AI continues to be integrated into educational practice, increasing attention will be needed to how these parallel efforts interact in everyday teaching and learning environments. Over time, the degree to which digital fluency, clinical judgment, and ethical reasoning are developed in coordination across generations is likely to shape the educational role of AI in medicine.
